# Supplementary material for: A semi-structured questionnaire survey of laboratory animal rehoming practice across 41 UK animal research facilities
Source: PLoS One. 2020 Jun 19;15(6):e0234922. doi: 10.1371/journal.pone.0234922 (PMC7304590; doi:10.1371/journal.pone.0234922)
Supplement: S1 Appendix — (DOCX) [file pone.0234922.s001.docx]

**Questionnaire on laboratory animal rehoming**

**Section 1 - Role and Background**

1. Name of research facility: ……………………………………………………………………………………………………………………………………………..
2. Your current role: ……………………………………………………………………………………………………………………………………………..
3. Main areas of research/education/conservation or other work undertaken by facility: ……………………………………………………………………………………………………………………………………………………………………………………………………………………………………………………………………………………………………………………………………………………………………………………………………………………………………………..

**Section 2 – Animals kept at facility**

1. Animals currently kept at facility (please tick all that apply)

Amphibians

Guinea pigs

Beagles

Rabbits

Other dogs

Cephalopods

Ferrets

Non-human primates

Goats

Fish

Other (please elaborate in the space below)

Common quail

Sheep

Mice

Other birds

Cattle

Rats

Hamsters

Pigs

Horses

Gerbils

Cats

1. Numbers kept of each type of animal at this time (please insert number kept next to the animal)

Amphibians

Goats

Rats

Hamsters

Sheep

Cephalopods

Gerbils

Common quail

Pigs

Cattle

Guinea pigs

Fish

Non-human primates

Other dogs

Horses

Sheep

Mice

Other birds

Other (please elaborate in the space below)

Cats

Rabbits

Beagles

Ferrets

**Section 3 - Rehoming policy**

**6a)** Does your facility have a rehoming policy?

Yes (complete questions 6b and 6c)

No (Complete questions 6d and 6e)

**6b)** Would it be available to this study?

Yes

No

**6c)** If yes, please email the policy.

**6d)** What are the facility’s reasons for not having a rehoming policy?

**6e)** Does the facility have plans to consider such a policy in the future?

**7)** Regardless of whether your facility has a formal policy in place, has your facility rehomed animals in the last 3 reporting years (2015-2017)?

Yes (Continue as normal with question 8)

No (Go to question 13)

**8)** Do you have records of the animals rehomed over the last 3 reporting years (2015-2017)?

Yes

No (Go to question 11)

1. Which types of animal has your facility rehomed over the last 3 reporting years? (2015-2017)

Amphibians

Guinea pigs

Beagles

Rabbits

Other dogs

Cephalopods

Ferrets

Non-human primates

Goats

Fish

Other (please elaborate in the space below)

Common quail

Sheep

Mice

Other birds

Cattle

Rats

Hamsters

Pigs

Horses

Gerbils

Cats

1. How many of each animal has your facility rehomed over the last 3 reporting years? (2015-2017) (please insert number rehomed next to the animal)

Amphibians

Guinea pigs

Beagles

Rabbits

Other dogs

Cephalopods

Ferrets

Non-human primates

Goats

Fish

Other (please elaborate in the space below)

Common quail

Sheep

Mice

Other birds

Cattle

Rats

Hamsters

Pigs

Horses

Gerbils

Cats

**Section 4 - Problems/risks encountered when rehoming**

1. Has your facility encountered any issues during the rehoming process?

Yes (go to question 12)

No (go to question 13)

1. Please tick all that apply

Some animals are unsuited to rehoming

New owners not able/wanting to keep animal

Costly process

Time consuming

Difficulty networking with relevant organisations (e.g. rehoming organisations/sanctuaries)

Attracted negative media attention

Difficulties finding suitable homes/sanctuaries

Other (please elaborate in space below)

………………………………………………………………………………………………………………………………………………………………………………………………………………………………………………………………………………………………………………................................................................................................................................................................................................................................................................................................................................................

**Section 5 - Opportunities presented by rehoming**

1. Which of these benefits does rehoming offer to your facility? (Please tick all that apply)

Shows a positive ethical stance

Good for staff morale

Expectation of future wellbeing of animal

No opportunities presented

Opportunity to develop networks with rehoming charities and the public

Other (please elaborate in space below)

……………………………………………………………………………………………………………………………………………………………………………………………………………………………………………………………………………………………………………………….......................................................................................................................................................................................................................................................................................................................................

**Section 6 - The rehoming process (please go to question 19 if your facility does not rehome animals).**

1. What measures does your facility take to determine whether or not an animal is suitable for rehoming?

……………………………………………………………………………………………………………………………………………………………………………………………………………………………………………………………………………………………………………………….......................................................................................................................................................................................................................................................................................................................................

1. Which of these measures does your facility take to prepare animals for rehoming (select N/A if group of animals is not kept):

|  | Training | Socialisation with people | Socialisation with other animals | Medical procedures (microchipping, neutering) | Exposure to new environments | None | N/A |
| --- | --- | --- | --- | --- | --- | --- | --- |
| Amphibians |  |  |  |  |  |  |  |
| Cephalopods |  |  |  |  |  |  |  |
| Fish |  |  |  |  |  |  |  |
| Birds |  |  |  |  |  |  |  |
| Rodents |  |  |  |  |  |  |  |
| Livestock |  |  |  |  |  |  |  |
| Horses |  |  |  |  |  |  |  |
| Cats |  |  |  |  |  |  |  |
| Dogs |  |  |  |  |  |  |  |
| Non-human primates |  |  |  |  |  |  |  |

1. What methods does your facility use to find homes for the animals?

Advertisements (online/newspapers/posters/flyers/mailing lists)

Animals are transferred to rehoming charities who undertake this process

Animals rehomed to staff/staff contact

Other (please elaborate in space below)

Word of mouth

……………………………………………………………………………………………………………………………………………………………………………………………………………………………………………………………………………………………………………………….......................................................................................................................................................................................................................................................................................................................................

1. Does your facility have criteria that potential owners must meet, such as prior experience of the species?

Yes (please elaborate in the space below)

No

……………………………………………………………………………………………………………………………………………………………………………………………………………………………………………………………………………………………………………………….......................................................................................................................................................................................................................................................................................................................................

1. Does your facility prepare owners?

Yes (please outline this process in the space below)

No

……………………………………………………………………………………………………………………………………………………………………………………………………………………………………………………………………………………………………………………….......................................................................................................................................................................................................................................................................................................................................

**Section 7 - Reasons for choosing not to rehome research animals (if you rehome animals, please go to section 8)**

1. If you are not rehoming animals, why is this? Please tick all that apply.

Monetary issues – lack of funding

Convenience – easier to euthanise

Fear of unwanted or negative media attention

Concern for the animal’s health if it were rehomed

High demand for animals means few are left to retire

Worries regarding being seen to support animal rights campaigners

Too stressful for the animal

Difficulties with transportation

Difficult to assess long-term health implications

Not aware that it is possible

Loss of control – once rehomed facility cannot be responsible for animal’s welfare

Never previously considered it

Other (please elaborate in the space below)

……………………………………………………………………………………………………………………………………………………………………………………………………………………………………………………………………………………………………………………….......................................................................................................................................................................................................................................................................................................................................

**Section 8 – Future interviews**

1. As part of my research project, I will be conducting a number of telephone and face-to-face interviews. These will aim to understand the rehoming process in a little more depth. Would you consider participating in an interview at a later date?

No

Yes (Thank you. Please insert below the email address you would prefer to be contacted on to arrange a time and date)

Email address: ………………………………………………………………………………………………………………………………………………………………………………………………………………………………………………………………………………………………………………………...

**Section 9 - Early access to results**

As you have completed the questionnaire, you are entitled to a summary of the results of the survey before the wider project results are released. Would you like a summary of the results?

Yes

No

If yes, please leave your email below. The email will not be used for any other purposes.

Email address: ………………………………………………………………………………………………………………………………………………………………………………………………………………………………………………………………………………………………………………………...
