# Supplementary material for: A semi-structured questionnaire survey of laboratory animal rehoming practice across 41 UK animal research facilities
Source: PLoS One. 2020 Jun 19;15(6):e0234922. doi: 10.1371/journal.pone.0234922 (PMC7304590; doi:10.1371/journal.pone.0234922)
Supplement: Attachment — Submitted filename: Response to reviewers.docx [file pone.0234922.s002.docx]

**Response to reviewers**

Thank you for the helpful comments.

1. The manuscript has now been changed to meet PLoS One’s style requirements.
2. The title has been changed in accordance with the editor’s comments, and is now informative about the study’s scope and methodology.
3. Figures have been changed to reflect that the data shown is only from facilities responses were obtained responses from.
4. The funding source has now been removed from the acknowledgements.
5. Additional information has been included in the methodology showing how data was gathered and analysed. A word document copy of the questionnaire has been included in the supporting information.
6. Suggestions that the data from this study be available on request have been removed due to ethical restrictions on data sharing. The cover letter has been revised to reflect these restrictions, and details the ethics committee that imposed them.

The length of the introduction and conclusion have also been shortened in accordance to comments made by the editor handling the manuscript. There have been additional modifications to the whole paper.

Additional revision:

1. Thank you again for useful considerations regarding data restrictions. Unfortunately, I cannot share the data for ethical reasons as consent was on the basis that the information gathered from participants would not be shared beyond the authors of this paper. I proceeded in this way as it is difficult to fully anonymise data in a small field. I hope this provides a satisfactory explanation.
