# Supplementary material for: A semi-structured questionnaire survey of laboratory animal rehoming practice across 41 UK animal research facilities
Source: PLoS One. 2020 Jun 19;15(6):e0234922. doi: 10.1371/journal.pone.0234922 (PMC7304590; doi:10.1371/journal.pone.0234922)
Supplement: Attachment — Submitted filename: Response to reviewers.docx [file pone.0234922.s004.docx]

*Thank you all for your very helpful comments.*

General: It is debatable whether the term euthanasia should be used when animals are killed in situations when it is not in their own interest. I recognize that the term euthanasia is predominant when referring to animals killed for research purposes, but I invite you to reflect on whether it is the best term in your paper. For example, you write "Euthanasia is undertaken for three primary reasons – 1) as a scientific  requirement, 2) to prevent avoidable suffering, or 3) for financial/logistical reasons". Critics would say that the term "euthanasia" only applies to 2) in the cited example. I understand that you are aware of the controversy and briefly mention it in the introduction with a reference to Kuře, J., Euthanasia: The" Good Death" Controversy in Humans and Animals. 2011: BoD–652 Books on Demand. Please consider changing to "killing" and justify your choice. *Thank you for your suggestion – this is really interesting. I have now used the word ‘killing’ throughout, but have explained the term euthanasia to relate to the act of putting an animal out of avoidable suffering.*

Table 1 I really appreciate the use of colour coding - please add that higher numbers are represented in more saturated colours. *Thank you, I have now added this in.*

Line 379 the owner is provided with appropriate housing and feeding **for the rehomed animal(s)**. *Thank you, I have changed in line with your comments.*

Line 155 Existing literature notes this as **an** essential next step in (or **the** essential). *Thank you! This has been changed accordingly.*

Lines 474-476 "there are challenges that come with rehoming to staff – an NVS explained how the rodents could not be rehomed to employees as staff might “acquire rodents from other sources” that are “microbiologically dirty […] which could present a risk of inadvertent delivery of disease”. This statement is a little puzzling as there is no obvious relation between rehoming rodents to staff and the same staff acquiring rodents from other sources. I assume that the relation may be that because microbiological contamination is considered a risk, staff are not allowed to keep rodents as pets, and therefore can't receive rehomed rodents. Do you know if this is the case? *Yes – that is the case. I have now made this clearer, and inserted: “Microbiological contamination is thus considered a risk, and therefore some facility staff cannot keep rodents as pets, including those from the laboratory.”*

Lines 496-500 and section Biosecurity Please provide a reference for that rehoming GA animals is not in itself illegal in the UK. *Thank you, I have now inserted a reference for the Home Office 2015 Advice Note that outlines that additional legislation may also apply to the rehoming of genetically altered animals.*

Figures 3-5: Please consider merging the 3 sets of graphs into one figure with 3 panels. *Thank you for the useful suggestion, I have now merged the 3 graphs into one figure.*

Reference list: Reference manager generated lists require careful manual checking. For example, "Office, H."  is not the right way to refer to the Home Office! There are also inconsistencies in whether journal titles are capitalized or not (they should be). *Thanks - this has now been addressed.*

**Reviewer #1**: Thank you for this very careful revision. You have implemented the reviewers' comments well. Please capitalize the word "owner" in the captions of Tables 4 and 5. *Thank you for your very helpful comments – tables 4 and 5 now have a capitalised ‘Owner’.*
 **Reviewer #2:** The authors were able to significantly improve the manuscript.

There is a remaining major concern about the discussion for me, which requires some additional work and consists of the following:
- Although it is true that not many papers exist on rehoming of research animals, there are some. Particularly with relevance to the section "finding the right home", I feel that it is appropriate to compare the practices to the shelter literature and investigated approach / recommended good practices. *Thank you for this very useful suggestion. I have added in some animal shelter literature and compared this to the findings of this research, mainly under the section “finding the right home”.*
- The authors report on the involvement of third party rehoming organisations. However, they should at least consider that these consist of volunteers who mean well, but who are rarely trained professionally for the duties they perform. This should be briefly discussed in relation to available literature on animal welfare volunteers. *Thanks for this – I have now reflected on this: “However, some of those that work at rehoming organisations are volunteers – and literature notes that these volunteers are not always taught the required skills to adequately train and socialise animals [32] In fact, only 12% of animal shelter employees across the US rated volunteers as well trained “to a great extent” [33]. Research also finds an irregular schedule of social contact with animals from volunteers and frequent changes in active volunteers [34, 35].”*
- The fact that an extrapolation was made from 2017 data to 2015 and 2016 (see lines 215-217 in the methods section) should be mentioned as a limitation to the study, as this is based on the assumption that numbers are similar in those three years (if there is a good reason to assume this, then it should be mentioned as well). *Thank you for this useful thought – I have now added “Finally, an extrapolation was made from 2017 Home Office data to the years 2015 and 2016 to calculate an approximate number of surplus animals across the 3 years. However, there is likely to be some variance in the proportions of surplus animals by species year by year that is not accounted for.”*

The remaining minor concerns:
Line 37: "this period" should be clarified. *Thank you, this period has now been clarified to between 2015-2017.*
Line 104: "desire" is perhaps a bit inappropriately anthropomorphic. Perhaps better to say something like "includes the degree to which the animal is provided with what it needs as well as wants" (or use "needs and wants" as these are commonly used terms in animal welfare science). *I have now added in “It includes the degree to which the animal is provided with its needs and wants”.*
Line 142: why use "sentient" here explicitly for companion animals? This implies that the subsequent species mentioned (rabbits, rats, guinea pigs and mice) are not sentient, which is not commonly accepted as they are vertebrates as well. *I have now removed the word sentient.*
Table 1 : mention the number of UK facilities in the column header of the second column. In the caption, explain the coding of the shading, i.e. darker shading indicates a higher number*. Thank you for your comments, I have now added in an explanation of the darker shading, and the total number of UK facilities.*
Lines 300 - 314: this feels repetitive (title is also almost identical to the one above). Why not introduce the percentages of animal groups in the text in the section above, and then refer to table 1 for details? *Thank you – I have removed table 2, and added in the text: “Although 94.15% of species kept in laboratories are rodents, they make up under a fifth (19.14%) of all animals known to be rehomed between 2015-2017. Conversely, birds, cats, dogs, horses, amphibians and agricultural animals constitute 80.86% of total species rehomed, despite making up just 5.84% of those kept (see Table 1 for more details). This is based on the following grouping: dogs (beagles and all other dog breeds), small mammals (rats, mice, gerbils, rabbits, hamsters, ferrets, guinea pigs), birds (common quail and all other birds), agricultural animals (cattle, sheep, pigs and goats), and cats, horses, amphibians and primates. Fish were excluded from this analysis due to one outlier facility having rehomed over 1200. There thus exists a preference for the rehoming of some species over others”*
Tables 3 and 4: captions should mention the total number of respondents. *I have now added in n=19 on the table caption.*
Lines 378-379: did the institution provide all the housing and food that the animal needed or did they provide some of it? It would be useful to have a specific example of what is meant. *Thank you – I have explained that it was generally initial food to help the animals acclimatise. I have also added in a quote “The animals are always released with items from their home cages and some diet. We inform the new owners it will take them quite a while to acclimatise and for the initial few days just to place their hands in the cage and let the animals come to them” to provide a specific example of what is meant.*
Lines 568-571: sentence not entirely clear, particularly the first part "this does not mean rehoming... kept in UK facilities". *Thank you – this had been modified to “Despite the clear impossibility of finding homes for all of the rodents and fish currently kept in UK facilities, there would be merit in understanding the drivers and processes of rehoming even small numbers of these species.”*
